# Astrocytes in preoptic area regulate acute nociception‐induced hypothermia through adenosine receptors

**DOI:** 10.1111/cns.14726

**Published:** 2024-05-07

**Authors:** Junke Jia, Ting Chen, Chang Chen, Tengxiao Si, Chenyi Gao, Yuanyuan Fang, Jiahui Sun, Jie Wang, Zongze Zhang

**Affiliations:** ^1^ Department of Anesthesiology, Zhongnan Hospital Wuhan University Wuhan China; ^2^ Wuhan Institute of Physics and Mathematics, Innovation Academy for Precision Measurement Science and Technology Chinese Academy of Sciences Wuhan China; ^3^ Institute of Neuroscience and Brain Diseases, Xiangyang Central Hospital Affiliated Hospital of Hubei University of Arts and Science Xiangyang China; ^4^ Shanghai Key Laboratory of Emotions and Affective Disorders, Shanghai Jiao Tong University School of Medicine Songjiang Hospital and Songjiang Research Institute Shanghai China

**Keywords:** adenosine receptor, astrocyte, hypothermia, pain, preoptic area

## Abstract

**Aims:**

The preoptic area (POA) of the hypothalamus, crucial in thermoregulation, has long been implicated in the pain process. However, whether nociceptive stimulation affects body temperature and its mechanism remains poorly studied.

**Methods:**

We used capsaicin, formalin, and surgery to induce acute nociceptive stimulation and monitored rectal temperature. Optical fiber recording, chemical genetics, confocal imaging, and pharmacology assays were employed to confirm the role and interaction of POA astrocytes and extracellular adenosine. Immunofluorescence was utilized for further validation.

**Results:**

Acute nociception could activate POA astrocytes and induce a decrease in body temperature. Manipulation of astrocytes allowed bidirectional control of body temperature. Furthermore, acute nociception and astrocyte activation led to increased extracellular adenosine concentration within the POA. Activation of adenosine A1 or A2A receptors contributed to decreased body temperature, while inhibition of these receptors mitigated the thermo‐lowering effect of astrocytes.

**Conclusion:**

Our results elucidate the interplay between acute nociception and thermoregulation, specifically highlighting POA astrocyte activation. This enriches our understanding of physiological responses to painful stimuli and contributes to the analysis of the anatomical basis involved in the process.

## Graphical abstract

Our findings revealed the integration of acute nociception and thermoregulation. Upon acute nociceptive stimulation, the activation of POA astrocytes led to hypothermia, mediated by adenosine A1 and A2A receptors.

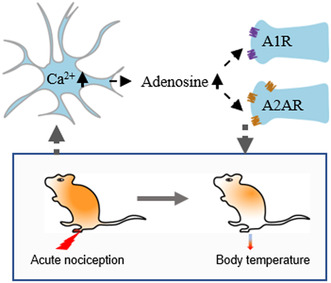

## 1 INTRODUCTION

The preoptic area of the hypothalamus (POA), a central hub for thermoregulation,^1^, ^2^, ^3^ has long been implicated in the pain process. Studies have demonstrated that both nonselective chemical activation of neurons within the POA and electrical stimulation in this region can elevate the pain threshold in rats.^4^, ^5^ Painful stimulation can trigger a cascade of physiological responses within the nervous system.^6^ However, the potential effects of acute pain on body temperature through POA remains poorly studied.

Emerging evidence has increasingly highlighted the functional significance of astrocytes.^7^, ^8^, ^9^ Nociception signals can be transmitted to astrocytes through immune activation and elevated central IL‐1 expression in response to peripheral inflammatory pain has been identified in astrocytes.^10^ Additionally, central astrocytes can be activated by the spontaneous activity of peripheral sensory nerves, contributing to pathological pain.^11^ The plasticity of astrocyte activity further underscores their dynamic role.^12^ In models of neuropathic pain, the central nervous system undergoes neuroglial network remodeling, characterized by increased reactive astrocyte in the amygdala.^13^ Studies indicate the involvement of central amygdala astrocytes in the emotional processing of chronic pain‐related stimuli.^14^ Astrocyte activation in the anterior cingulate cortex is also implicated in the affective component of inflammatory pain^15^ and aversive memory related to visceral pain.^16^ Our speculation centered on the potential role of POA astrocytes in mediating the effects of acute nociception on body temperature. To investigate this, we specifically monitored astrocyte activity during acute nociception and manipulated astrocytes within the POA to discern their roles in thermoregulation.

Neurotransmitters serve as chemical messengers and play a crucial role throughout the nervous system.^17^ The activation of astrocytes has a widespread impact on extracellular neurotransmitters. Evidence from hippocampal, olfactory bulb, and thalamic slices suggests that astrocytes release glutamate to modulate neuronal plasticity.^18^ The activation of astrocytes can also increase the levels of d‐serine, glycine, and adenosine in the glial environment, which then act on corresponding receptors, regulating neuronal excitability.^19^, ^20^, ^21^ Neurons influenced by astrocyte activation can also manifest changes in signal transmission. Studies have shown that the activated astrocytes act on presynaptic neurons, thereby amplifying neural transmission indirectly.^22^, ^23^ Consequently, we try to explore the potential neurotransmitter that might play a role in the temperature reduction process triggered by astrocytes activation. Activation of central adenosine receptors has been recognized as crucial in inducing and sustaining hypothermia when challenged by environment.^24^ By combining the chemical–genetic designed receptor exclusively activated by designed drug approach with a genetically encoded G protein–coupled receptor (GPCR)–activation‐based sensor for adenosine (GRAB_Ado_),^25^ we aimed to assess the impact of astrocyte activation on extracellular adenosine concentration within the POA.

Our study endeavors to uncover the previously uncharacterized relationship between nociception and thermoregulation. We demonstrated that acute nociceptive stimuli could lead to hypothermia, with POA astrocytes playing a pivotal role in this process. Upon activation, these astrocytes elevate extracellular adenosine levels and reduce body temperature by acting on adenosine A1 and A2A receptors. Thus, our research expands our understanding of pain‐related physiological responses and contributes to elucidating their anatomical basis.

## 2 METHODS

### 2.1 Animals

All animal procedures were approved by the Animal Ethics Committee of Wuhan University of Zhongnan Hospital (ZN2023224), and adhered to the ARRIVE guidelines. C57BL/6J mice (male, 8–14 weeks old) were purchased from Liaoning Changsheng Biotechnology Co. (Liaoning, China).

The mice had ad libitum access to food and water and were maintained on a natural 12‐h light/12‐h dark cycle (with lights on from 8 a.m. to 8 p.m.). The room was maintained at a temperature of 22–24°C and a humidity range of 40%–60%.

### 2.2 Animal model

#### 2.2.1 Capsaicin and formalin injections

On the study day, 20 μL capsaicin (1 mg/mL)^26^, ^27^ or 5% formalin (5% v/v prepared from a 40% stock formaldehyde solution)^28^ was injected into the plantar surface of the hind paws of mice using an insulin syringe while under brief isoflurane anesthesia. Phosphate‐buffered saline (PBS) was administered as the control.

#### 2.2.2 Surgery

Penetrating wound and skin incision models were created based on previous research.^29^ Mice were anesthetized with 1.5% isoflurane throughout the surgery. The hind legs were shaved, and incisions were made 2 mm distal to the stifle joint. For the penetrating wound, a 5‐mm incision was made through the skin and subcutaneous tissue. A surgical blade was then used to create a deep penetrating stab wound through the incision. Superficial skin incisions were made without a penetrating stab wound. The incised skin was closed with sutures. Sham surgery involved anesthetizing and shaving without making any incisions or stab wounds.

#### 2.2.3 Temperature monitoring

Rectal temperature was monitored using a Physio Suite monitor (Kent Scientific, USA). Each temperature data point was obtained by inserting the temperature probe into the mouse rectum for a minimum of 10 min. Specifically, for temperature measurement intervals not exceeding 10 min, continuous temperature monitoring was employed, with the temperature probe left in the rectum without removal. When the temperature measurement interval exceeded 10 min, the probe was removed after recording one temperature reading. It was reinserted at least 10 min before the next recording to allow the mice to adapt, minimizing the impact of stress on body temperature.

### 2.3 Stereotaxic surgeries and virus injection

The mice were deeply anesthetized with ketamine (dissolved in 1% pentobarbitone, intraperitoneal injection, 40 mg/kg, Jiangsu Hengrui Pharmaceuticals Co., Ltd., China) and then secured in a stereotaxic apparatus (RWD, China). For virus injection, the skull above the target area was carefully thinned using a dental drill and subsequently removed. The virus was delivered through a pulled glass capillary with a diameter of 10–15 mm connected to a Nanofil syringe (WPI, USA) and a pump (Stoelting, USA) mounted on the stereotactic frame.

For astrocytic targeting of hM3D(Gq) and GCaMP7b, as well as neuronal targeting of GRAB_Ado_, virus vectors were injected into the POA at the following coordinates: anterior–posterior (AP), 0 mm; medial–lateral (ML), + or −0.3 mm; and dorsal–ventral (DV), −5.15 mm, from bregma. Postinjection, the capillary remained in place for an additional 10 min to ensure proper virus diffusion before it was slowly withdrawn. Following the surgery, the mice were placed on a heated pad to facilitate recovery from anesthesia.

For the selective chemogenetic activation of astrocytes in the POA, mice received unilateral injections of adeno‐associated virus (AAV)2/5‐GfaABC1D‐hM3D(Gq)‐mCherry (titer: 5 × 10^12^ vector genomes (v.g.)/mL, 250 nL) and AAV2/5‐GfaABC1D‐mCherry (titer: 2.98 × 10^12^ v.g./mL, 250 nL). Injections of AAV2/5‐GfaABC1D‐CaMP7b (titer: 3 × 10^12^ v.g./mL, 250 nL) were administered for Ca^2+^ imaging of astrocytes. For extracellular adenosine recording, injections of AAV2/9‐hSyn‐Ado 1.0 (titer: 2 × 10^12^ v.g./mL, 250 nL) and AAV2/9‐hSyn‐mut.sensor (titer: 2.66 × 10^12^ v.g./mL, 250 nL) were administered.

For fiber optic recording, fiber optics (core diameter: 200 μm, Inper, China) were implanted immediately after viral injection. The coordinates for fiber optic implantation were as follows: AP, 0 mm; ML, + or − 0.3 mm; and DV, −4.95 mm. In cannula infusion experiments, guide cannulas (6 mm length, RWD, Shenzhen, China) were implanted into the POA. The coordinates for cannula implantation were as follows: AP, 0 mm; ML, 0 mm; and DV, −4.75 mm. Imaging, fiber optic recordings, and behavioral tests were conducted 3 weeks after virus expression. Verification of the optic fiber and guide cannula placement was conducted at the conclusion of the experiments, and data from properly injected mice were utilized for statistical analyses.

### 2.4 Drug administration

All drugs were dispensed on the spot. Clozapine‐N‐oxide (CNO) (Tocris, 4936), fluorocitric acid (FC) (Sigma, F9634), DCPCX (MCE, HY‐100937), SCH58261 (Selleck, S8104), and CGS 21680 (Selleck, S2153) were prepared as stock solutions in dimethyl sulfoxide (DMSO) and subsequently diluted to their final concentrations in sterile 0.9% saline. CCPA (Sigma, C7938) was dissolved directly in sterile 0.9% saline and further diluted to the desired concentration. The final concentrations employed were 100 mM for FC and 10 mM for CCPA, DCPCX, SCH58261, and CGS21680. For intraperitoneal (i.p.) injection, rats were administered CNO at 3 mg/kg body weight or the corresponding vehicle. In the case of Ca^2+^ and GRAB_Ado_ imaging, 10 mM CNO was applied to the slices by dissolving it in the perfused artificial cerebrospinal fluid (ACSF). Intracerebral drug delivery was facilitated through previously implanted infusion cannulas. On the day of the experiment, the internal cannulas, extending 0.5 mm beyond the ends of the guide cannulas, were inserted, and drugs (500 nL/side) were infused. The control groups received an equivalent amount of DMSO dissolved in sterile 0.9% saline or an equivalent volume of sterile 0.9% saline.

### 2.5 Slice preparation

For Ca^2+^ and GRAB_Ado_ imaging, mice were anesthetized and decapitated. Coronal slices (300 μm thick) were prepared using a vibratome (Leica VT1000S) in ice‐cold sucrose cutting solution, which consisted of the following (in mM): 30 NaCl, 4.5 KCl, 1.2 NaH_2_PO_4_, 26 NaHCO_3_, 10 d‐glucose, 1 MgCl_2_, and 194 sucrose. Subsequently, the slices were transferred to an incubation chamber filled with oxygenated ACSF containing the following (in mM): 124 NaCl, 4.9 KCl, 1.2 KH_2_PO_4_, 2.0 MgSO_4_, 2.0 CaCl_2_, 24.6 NaHCO_3_, and 10 D‐glucose. The slices were allowed to recover at 36°C for a minimum of 30 min.

### 2.6 Slice Ca^2+^ and GRAB_Ado_ imaging

Ca^2+^ and GRAB_Ado_ imaging of POA was conducted using a confocal laser scanning microscope (Leica TCS SP8) equipped with a 25× water‐immersion objective lens (NA = 0.95) and the 488 nm laser line was employed. Images were scanned at a rate of 0.5–1 frame per second. Subsequently, the Ca^2+^ and GRAB_Ado_ signals were processed using ImageJ (NIH) and measured as the fluorescence change over the baseline (ΔF/F).

### 2.7 Immunohistochemistry

Mice were deeply anesthetized with isoflurane and transcardially perfused with cold PBS, followed by 4% paraformaldehyde (PFA). The brains were isolated from the skull and fixed in 4% PFA overnight. Subsequently, cryoprotection was performed using a 30% sucrose solution for 24–48 h at 4°C, and coronal sections with a thickness of 40 μm were obtained using a cryostat microtome (Thermo Fisher, CRYOSTAR NX50). The free‐floating sections were rinsed in PBS (5 times, 5 min each) and then incubated in a blocking solution (consisting of 5% normal donkey serum or 10% normal goat serum and 0.3% Triton X‐100 in PBS) for 1 h at 37°C. This was followed by incubation with primary antibodies, including Rabbit anti‐c‐fos (2250, Cell Signaling, 1:1000), Rat anti‐c‐fos (226017, SYSY, 1:1000), Rabbit anti‐A1R (ab82477, Abcam, 1:100), Rabbit anti‐A2AR (a1587, Abclonal, 1:100), Goat anti‐GFAP (ab53554, Abcam, 1:1000), and Rabbit anti‐NeuN (ab177487, Abcam, 1:1000), overnight at 4°C. The sections were washed with PBS (5 times, 5 min each) and then incubated with secondary antibodies, including Donkey anti‐Rabbit conjugated to Alexa Fluor 488 (711r‐545‐152, Jackson ImmunoResearch, 1:300), Donkey anti‐Rabbit Cy3 (711‐165‐152, Jackson ImmunoResearch, 1:500), Donkey anti‐Goat Alexa Fluor 488 (705‐545‐147, Jackson ImmunoResearch, 1:300), Donkey Anti‐Goat Cy3 (705‐165‐147, Jackson ImmunoResearch, 1:500), Goat anti‐Rabbit Alexa Fluor 647 (111‐607‐008, Jackson ImmunoResearch, 1:500), and Goat anti‐Rat Alexa Fluor 488 (A11006, Thermo Fisher Scientific, 1:500) for 1 h at 37°C. The sections were washed with PBS, stained with 4′,6‐diamidino‐2‐phenylindole (DAPI) for 12 min, mounted with 70% glycerol and imaged using a confocal microscope (Leica, TCS SP8, Buffalo Grove, IL) or a virtual microscopy slide‐scanning system (Olympus, VS. 120, Tokyo, Japan).

### 2.8 Statistical analysis

Statistical analysis was performed using SPSS 20 (IBM, Armonk, NY) and graphed using Prism 9.0 (GraphPad, La Jolla, CA). Data are presented as mean ± SEM. Each *n* indicates the number of biologically independent replicates. Sample sizes were chosen on the basis of previous experience with similar models. To examine the distribution of the data, the Shapiro–Wilk normality test was used. One‐way analysis of variance with Tukey post hoc tests were used for multiple comparisons. The significance of differences between two groups was tested using an unpaired two‐tailed *t*‐test. Variables that do not exhibit a normal distribution were analyzed using Mann–Whitney *U*‐test or Kruskal–Wallis test. Significance was considered at *p* < 0.05.

## 3 RESULTS

### 3.1 Acute nociception could activate POA and reduce body temperature

**FIGURE 1:**
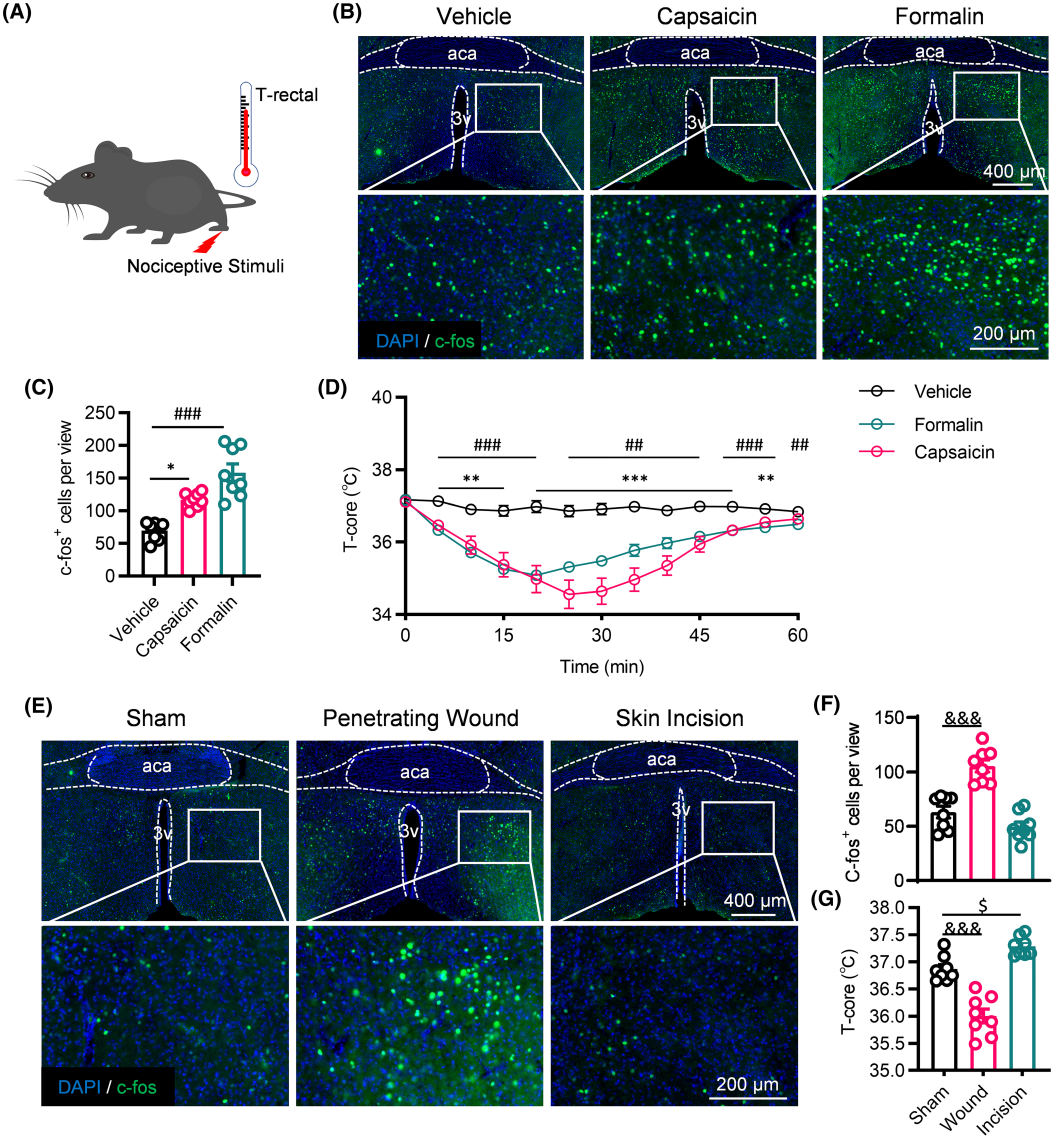
Acute nociception could activate POA and reduce body temperature. (A) Diagram illustrating the acute nociception and monitoring of body temperature. (B, C) Immunohistochemical staining and statistical data of c‐fos‐positive cells in the POA following intraplantar injection of vehicle, capsaicin and formalin (*n* = 8 brain slices from 6 mice). (D) Variation in body temperature following injection of vehicle, capsaicin, and formalin (*n* = 6). (E, F) Immunohistochemical staining and statistical data of c‐fos positive cells in the POA of sham, penetrating wound, and skin incision group (*n* = 8 brain slices from 6 mice). (G) Statistical data of body temperature of the mice from sham, penetrating wound, and skin incision group (*n* = 8). **p < 0.01 and ***p < 0.001 vehicle versus capsaicin group; ^##^ *p* < 0.01 and ^###^ *p* < 0.001 vehicle versus formalin group; ^&&&^ *p* < 0.001 sham versus penetrating wound group; ^$^ *p* < 0.05 sham versus skin incision group.

To ascertain the effect of acute nociception on body temperature, we monitored the core temperature through rectal measurements during acute nociceptive stimulation (Figure 1A). C‐fos staining confirmed the POA's reactivity to acute nociceptive stimulation induced by capsaicin and formalin (Figure 1B,C). Following capsaicin injection, we observed a gradual and substantial decline in the mice's body temperature, reaching its lowest point approximately 30 min postinjection (Figure 1D). Similarly, intraplantar administration of formalin led to a decreased body temperature, reaching its minimum around 20 min (Figure 1D).

Although intraplantar injections of capsaicin and formalin are established models for acute nociception, for higher translational potential, we used the hind limb deep penetrating wound and superficial skin incision^29^ to assess their effects on body temperature. We monitored body temperature 30 min after surgery and examined c‐fos‐positive cells in the POA. The data revealed significant activation of the POA due to the penetrating wound (Figure 1E,F) and a decrease in body temperature (Figure 1G). Conversely, the skin incision showed no impact on c‐fos expression in the POA (Figure 1E,F) and did not significantly alter body temperature (Figure 1G). The study indicated that deep pain and cutaneous pain might respectively inhibit and excite the sympathetic nervous system.^6^ The tissue origin or the intension of pain may exert differing effects on body temperature.

### 3.2 Acute nociception activated POA astrocytes

**FIGURE 2:**
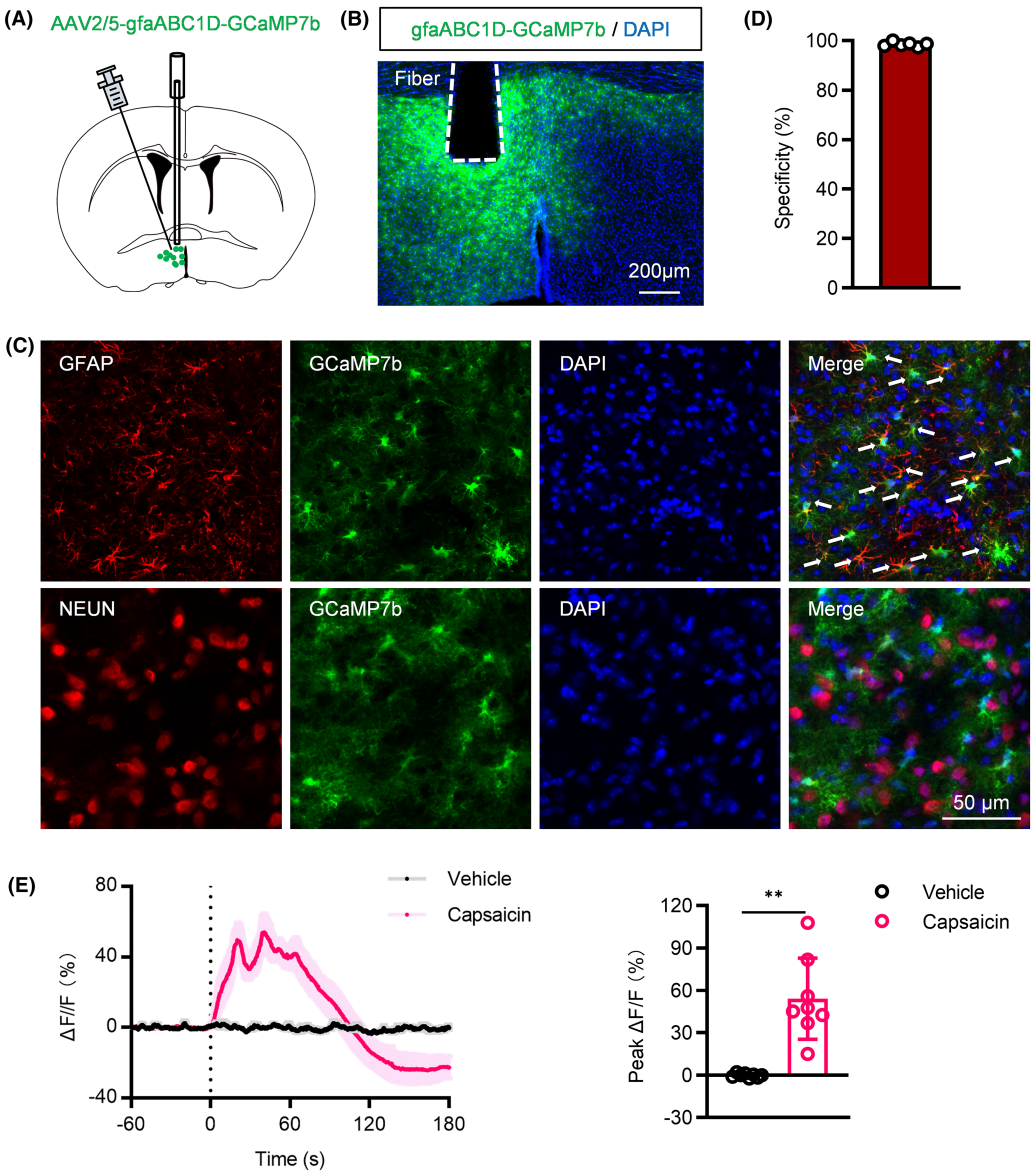
Activation of POA astrocyte following intraplantar injection of capsaicin. (A) Schematic representation of fiber optic recording. (B) Representative image displaying AAV‐gfaABC1D‐GCaMP7b expression and fiber location in the POA. (C) Immunohistochemical verification of GCaMP7b expression in POA astrocytes. (D) Histogram depicting the percentage of GFAP‐positive cells among GCaMP7b‐positive cells (*n* = 6 brain slices from 4 mice). (E) Average calcium activity and peak ΔF/F, indicating a significant increase in Ca^2+^ signals in response to capsaicin compared to vehicle‐treated mice (*n* = 8). **p < 0.01.

To investigate the dynamics of astrocytic [Ca^2+^]i following intraplantar capsaicin injection in the POA, we employed in vivo fiber photometry recording using the genetically encoded Ca^2+^ indicator, GCaMP7b^30^ (Figure 2A,B). We achieved efficient and selective expression of GCaMP7b in astrocytes within the POA by locally injecting a viral vector expressing GfaABC1D‐GCaMP7b (Figure 2C,D). Upon capsaicin administration, there was a notable increase in [Ca^2+^]i levels within POA astrocytes, a response not observed with the vehicle injection (Figure 2E). Moreover, to exclude capsaicin's nonspecific effects, we monitored [Ca^2+^]i levels in POA astrocytes following intraperitoneal injection, also serving as thermal stimulation,^1^ revealing no significant change (data not shown).

### 3.3 Manipulating POA astrocytes can regulate body temperature

**FIGURE 3:**
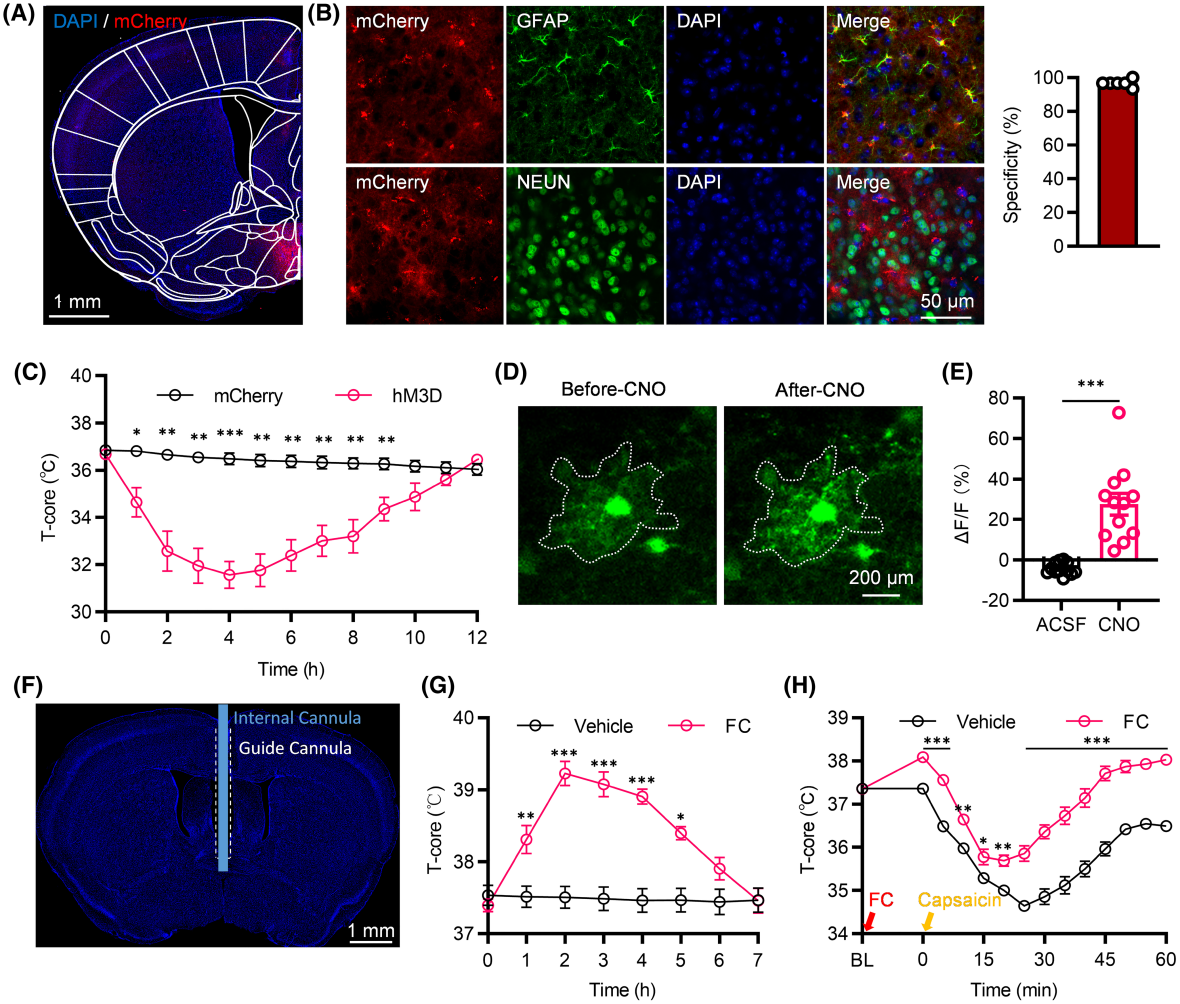
Manipulating POA astrocytes can regulate body temperature. (A) Representative image illustrating AAV‐gfaABC1D‐hM3D(Gq)‐mCherry expression in the POA. (B) Immunohistochemical verification of hM3D expression in POA astrocytes and histogram presenting the percentage of GFAP‐positive cells among hM3D‐positive cells (*n* = 6 brain slices from 4 mice). (C) Effects of chemogenetic activation of POA astrocytes on body temperature (*n* = 6). (D) Sample images depicting Ca^2+^ signals of POA astrocyte before and after CNO application. (E) Population average from astrocytes treated with ACSF or CNO (*n* = 12 cells from 3 mice). (F) Representative images displaying the position of cannula. (G) Impact of POA astrocyte inhibition via FC microinjection on body temperature (*n* = 6). (H) Effects of pretreatment with FC for 1 h on capsaicin–induced hypothermia (*n* = 6). *p < 0.05, **p < 0.01, ***p < 0.001. BL, baseline.

We investigated the thermoregulatory impact of selectively stimulating astrocytes in the POA with GfaABC1D‐hM3D(Gq) (Figure 3A), which were efficiently and selectively expressed in the astrocytes (Figure 3B). Control groups consisted of mice injected with the virus expressing GfaABC1D‐mCherry. Three weeks later, we administered the hM3D(Gq) ligand, CNO, intraperitoneally in the mice to activate the astrocytes, leading to a notable reduction in body temperature (Figure 3C). To validate that hM3D(Gq) evokes astrocyte activation, we performed Ca^2+^ imaging of POA astrocytes labeled with GCaMP7b (Figure 3D). CNO (10 mM), but not ACSF, application triggered robust Ca^2+^ responses in hM3D(Gq)‐expressing astrocytes (Figure 3E).

In contrast, we inhibited astrocyte activity by injecting FC, a widely used astrocyte metabolism inhibitor,^31^, ^32^, ^33^ into the POA (Figure 3F). Microinjection of FC effectively elevated the body temperature of the mice (Figure 3G). Furthermore, we pre‐injected FC through the cannula into the POA to inhibit astrocytes. One hour later, we administered capsaicin via plantar injection and measured the body temperature. Although it remained higher than that in controls, pre‐inhibition of POA astrocytes did not entirely prevent the decrease of body temperature (Figure 3H). Activation of POA astrocytes may be sufficient but not necessary for acute nociception‐induced hypothermia.

### 3.4 Acute nociception triggered release of adenosine in the POA

**FIGURE 4:**
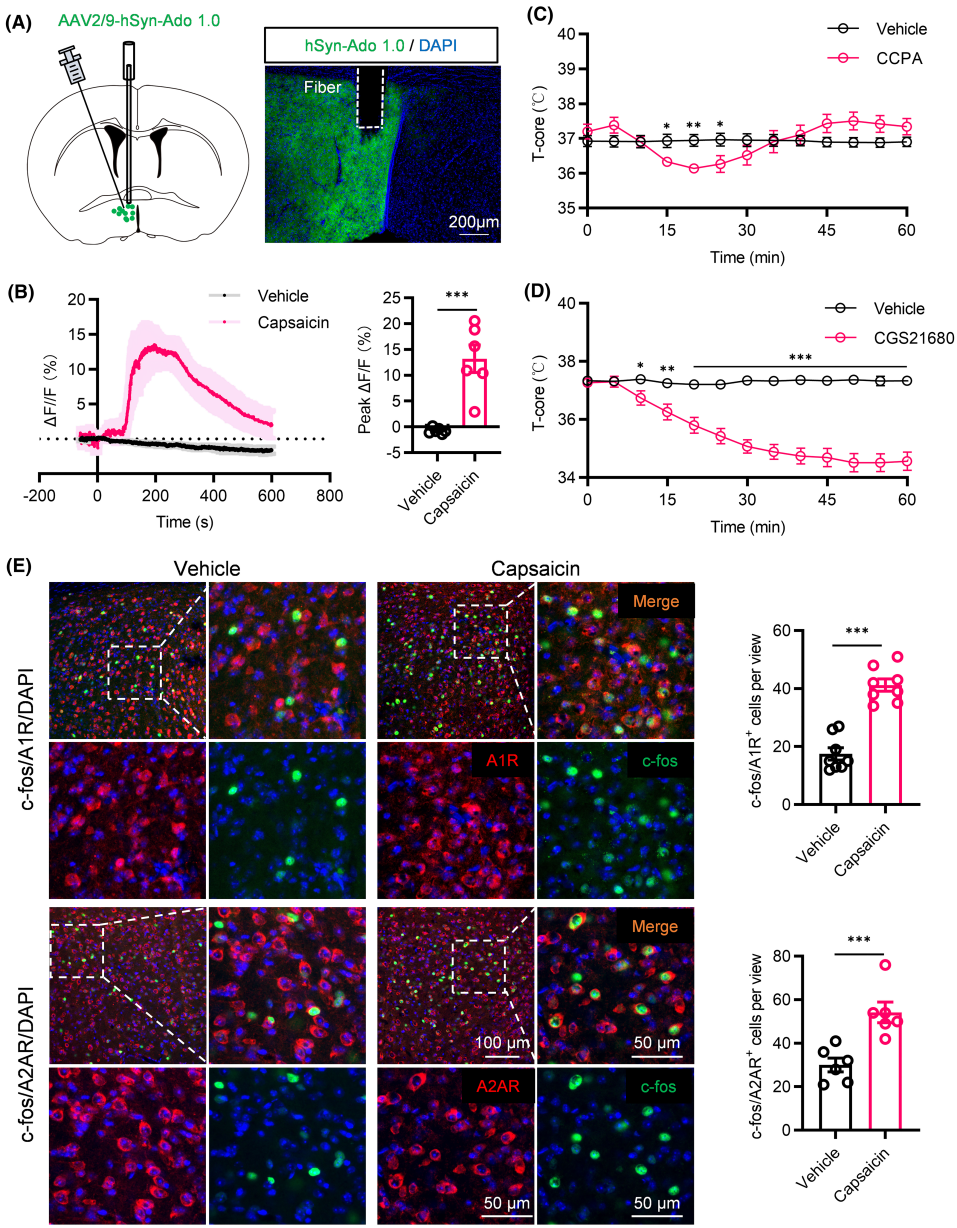
Effects of capsaicin intraplantar injection on adenosine release and modulation of hypothermia via adenosine A1 and A2A receptors. (A) Schematic representation of fiber optic recording (left) and representative image of AAV‐hSyn‐Ado 1.0 expression in the POA (right). (B) Average GRAB_Ado_ activity and peak ΔF/F illustrating that extracellular adenosine signals were increased significantly in response to capsaicin compared to vehicle mice (*n* = 6). (C) Body temperatures of vehicle and A1R agonist‐treated mice. (D) Body temperatures of vehicle and A2AR agonist‐treated mice. (E) Vehicle and capsaicin‐induced c‐fos colocalized with the A1R (upper) (*n* = 8 brain slices from 6 mice) and A2AR (lower) (*n* = 6 brain slices from 6 mice) in the POA. *p < 0.05, **p < 0.01, ***p < 0.001.

We evaluated extracellular adenosine concentration dynamics in the POA during capsaicin‐induced pain using the GRAB_Ado_ sensor.^25^ Injecting an AAV expressing GRAB_Ado_ into the POA, we monitored fluorescence signal changes via fiber photometry (Figure 4A). Post‐capsaicin injection, we observed a notable rise in extracellular adenosine levels compared to the vehicle (Figure 4B).

Our subsequent focus was to identify the receptor(s) responsible for the nociception‐induced reduction in body temperature. Adenosine exerts its effects through the activation of a family of GPCRs known as A1, A2A, A2B and A3 receptors.^34^ Through microinjections of adenosine receptor agonists into the POA, we found that the selective adenosine A1 receptor (A1R) agonist (CCPA) (Figure 4C) and A2A receptor (A2AR) agonist (CGS21680) (Figure 4D) notably decreased body temperature. This effect was not observed with A2B and A3 receptors (data not shown). Additionally, capsaicin intraplantar injection activated a greater number of cells expressing adenosine A1R and A2AR in the POA (Figure 4E). These findings suggested the involvement of adenosine A1R and A2AR in the acute nociception‐induced hypothermia.

### 3.5 Modulation of extracellular adenosine release by astrocytes in POA

**FIGURE 5:**
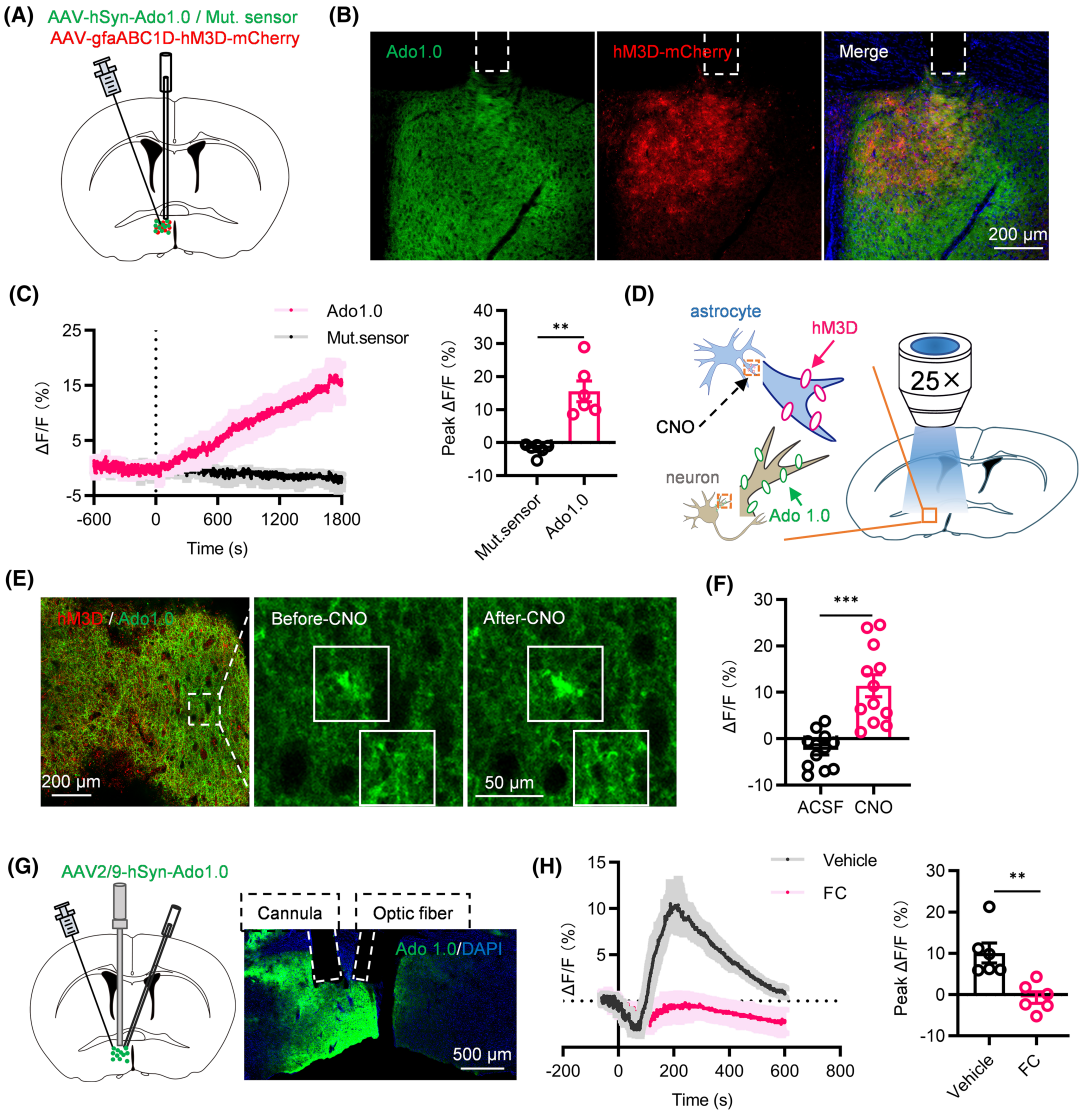
Regulation of adenosine release by astrocytes. (A) Schematic representation of fiber optic recording. (B) Representative image demonstrating AAV‐gfaABC1D‐hM3D and AAV‐hSyn‐Ado 1.0 expression in the POA. (C) Average activity of GRAB_Ado_ and Mutant sensor and peak ΔF/F, indicating a significant increase in extracellular adenosine signals following astrocyte activation (*n* = 6). (D) Schematic of viral injection and recording configuration under confocal microscopy. (E) Expression of hM3D(Gq) and GRAB_Ado_ in POA (left), along with sample images depicting extracellular adenosine signals before and after CNO application. (F) Response of GRAB_Ado_ in the POA treated with ACSF or CNO (*n* = 12 regions from 3 mice). (G) Schematic illustration (left) and representative image (right) displaying the positioning of the cannula and optic fiber. (H) Average GRAB_Ado_ activity and peak ΔF/F, demonstrating that compared to vehicle, FC pretreatment significantly inhibited extracellular adenosine signals in response to capsaicin (*n* = 6). **p < 0.01, ***p < 0.001.

To detect the relationship between astrocyte activation and the adenosine levels in the POA, we chemogenetically activated astrocytes and measured adenosine levels using fiber photometry. We achieved this by locally injecting AAVs expressing hM3D(Gq) and GRAB_Ado_ (Figure 5A,B). Chemogenetic activation of astrocytes through CNO i.p. administration significantly increased extracellular adenosine levels (Figure 5C), suggesting the involvement of astrocytes in modulating adenosine levels during nociceptive stimulation. To confirm that astrocyte activation prompts adenosine release, we performed GRAB_Ado_ imaging on POA astrocytes (Figure 5D). Application of CNO (10 mM), but not ACSF, evoked robust GRAB_Ado_ responses in hM3D(Gq)‐expressing astrocytes (Figure 5E,F).

To investigate the necessity of astrocyte activation in driving the increase in extracellular adenosine concentration following nociceptive stimulation, we administered microinjections of FC into the POA 1 h before intraplantar capsaicin injection (Figure 5G). Mice injected with FC showed a significantly reduced adenosine elevation compared to those injected with the vehicle upon intraplantar capsaicin stimulation (Figure 5H).

### 3.6 Essential role of A1R and A2AR in astrocyte‐induced hypothermia in POA

**FIGURE 6:**
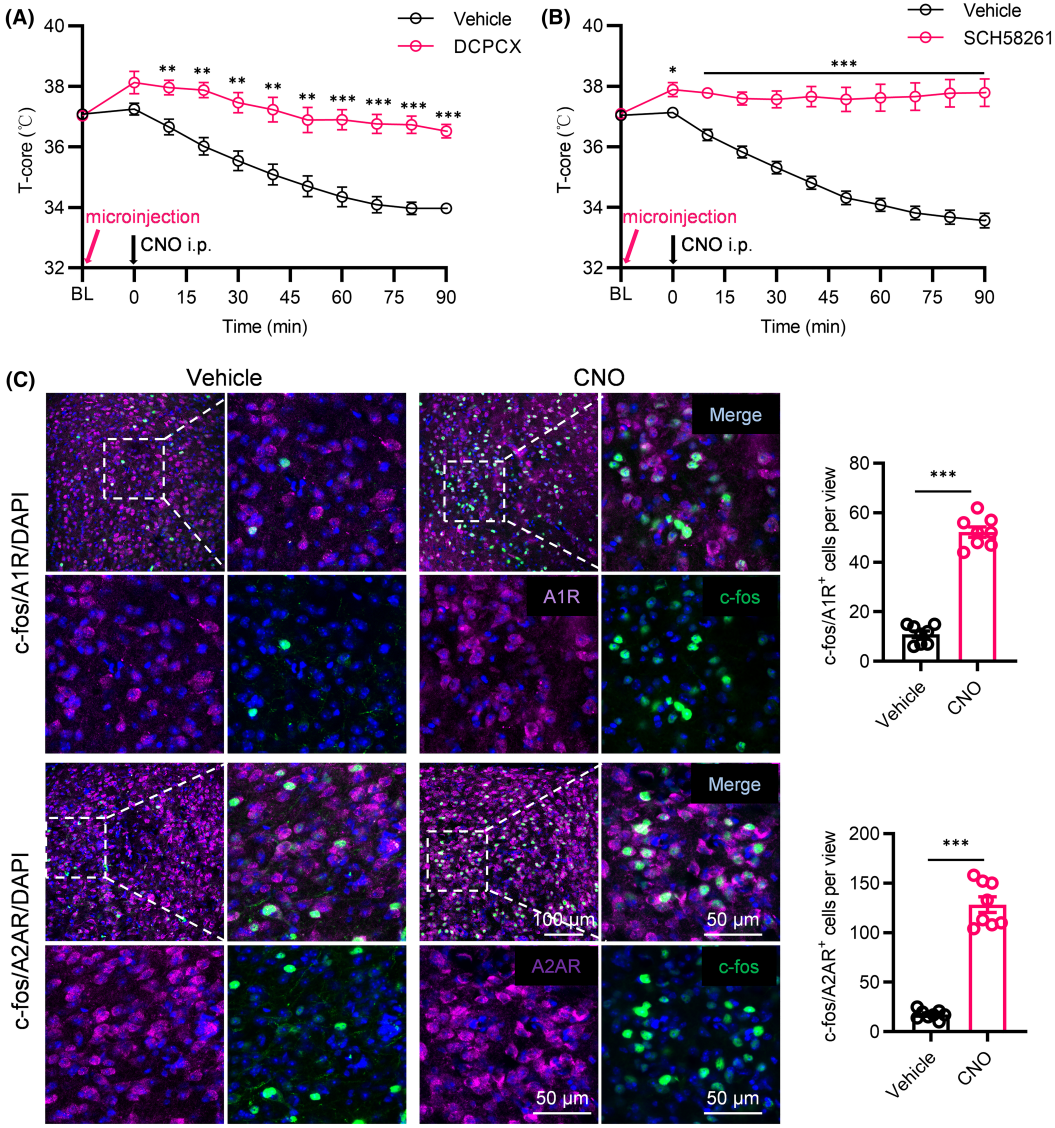
Requirement of A1R and A2AR for astrocytes in modulating body temperature. (A) Effects of chemogenetic activation of astrocyte on body temperature after 30 min of pre‐administration of an A1R antagonist in the POA region. (B) Effects of chemogenetic activation of astrocyte on body temperature after 30 min of pre‐administration of an A2AR antagonist in the POA region. (C) CNO and vehicle‐induced c‐fos colocalized with the A1R (upper) and A2AR (lower) in the POA of hM3Dq mice (*n* = 8 brain slices from 6 mice). *p < 0.05, **p < 0.01, ***p < 0.001.

To further confirm the involvement of adenosine receptors in astrocyte activation‐induced hypothermia, after AAV‐GfaABC1D‐hM3Dq injection, we embedded the cannula into the POA of mice. Thirty minutes after separate microinjections of the specific A1R antagonist, DCPCX, or the specific A2AR antagonist, SCH58261, into the POA via the cannulas, the mice received CNO i.p. to activate astrocytes. Microinjection of DCPCX (Figure 6A) or SCH58261 (Figure 6B) significantly mitigated the hypothermia induced by the chemogenetic activation of astrocytes in the POA compared to vehicle‐treated mice. Immunofluorescence co‐labeling also revealed a notable increase in the activated cells expressing A1R and A2AR in the POA following the chemogenetic activation of POA astrocytes (Figure 6C). These results strongly indicated the essential role of A1R and A2AR in the ability of astrocytes in the POA to induce hypothermia.

## 4 DISCUSSION

In this study, we unveiled a previously uncharacterized phenomenon wherein acute nociception could lead to a decrease in body temperature. Our investigation highlighted the pivotal role of POA astrocytes in this process. Upon activation, POA astrocytes demonstrated a significant capacity to induce hypothermia. While, inhibiting astrocytes resulted in an elevated body temperature. Furthermore, our findings suggested the involvement of endogenous adenosine in mediating the function of astrocytes in the POA.

Intraplantar injections of capsaicin and formalin, as well as surgery, represent reliable models for pain stimulation. The formalin model encompasses a biphasic pain response, with the initial phase (0–15 min) reflecting acute peripheral hypersensitivity and the second phase (15–60 min) associated with the maintenance of pain through central sensitization.^28^, ^35^ Due to the relatively intricate mechanism of formalin‐induced pain and potential confounding factors such as bleeding during surgery, we chose to further investigate the mechanism of hypothermia induced by noxious stimuli using the capsaicin model.

Painful stimuli are initially detected by peripheral nociceptive neurons, transduced into neuronal electrical activity, and subsequently transmit signals to the central nervous system through the release of a variety of neurotransmitters to produce appropriate sensory percepts and physical response.^36^ Our study demonstrated that acute nociception could induce a remarkable decrease in body temperature. Hypothermic treatment of nerves effectively reduces the metabolism, electrogenesis, and ionic activity of nerve tissue.^37^ Cryotherapy has also been observed to induce antinociceptive effects^38^, ^39^, ^40^, ^41^ by leveraging the negative temperature‐dependent neural activities. The acute nociception‐induced hypothermia may serve as a protective response to painful stimuli.

Thermoregulation is orchestrated by intricate neural circuits. Various neural populations within the POA have been identified as triggers of hypothermia. These populations include subsets of glutamatergic neurons expressing leptin receptors in the median POA (MnPO),^42^ neurons expressing brain‐derived neurotrophic factor and pituitary adenylate cyclase‐activating polypeptide in the MnPO and medial POA,^1^ and GABAergic neurons in the ventral lateral POA.^43^ The involvement of the hypothalamus in acute pain has been well‐documented through micro‐stimulation and imaging studies in humans.^44^, ^45^ Our c‐fos immunostaining results underscore the involvement of the POA in acute noxious stimulation. The role of astrocytes in the central nervous system has garnered increased attention. Astrocyte activation manifests as an elevation in intracellular Ca^2+^ ([Ca^2+^]i) levels.^46^, ^47^ Furthermore, we demonstrated the immediate activation of POA astrocytes following intraplantar injection of capsaicin. Manipulation of these astrocytes resulted in changes of body temperature, affirming the role of POA astrocytes in nociception‐induced hypothermia.

It is notable that inhibiting astrocytes did not prevent the temperature decrease following noxious stimuli in our study, suggesting the involvement of pathways independent of astrocytes in this process. Studies indicate that astrocytes only influence approximately 40% of synapses,^48^, ^49^ leaving the majority of neurons capable of independent function. As mentioned earlier, multiple neuronal nuclei in the POA can respond to environmental stimuli, resulting in a decrease in body temperature. Therefore, it cannot be ruled out that there are neurons directly involved in the temperature decrease induced by noxious stimuli.

Adenosine is recognized for its stress‐induced release and homeostatic regulatory functions, playing a significant role in cell and tissue protection during injury.^50^ Central adenosine receptor activation has been associated with a state resembling hibernation in animals, akin to the considerable reduction in body temperature observed in our study.^51^, ^52^ Within the hypothalamus, purine signaling has long been implicated in body temperature regulation.^53^ In our study, the activation of POA astrocytes could elevate extracellular adenosine levels. The thermoregulatory role of POA astrocytes may depend on the adenosine signaling. Although studies have demonstrated adenosine as a crucial neurotransmitter released by astrocytes,^54^, ^55^, ^56^ there are also indications that the elevated adenosine in the basal forebrain and hippocampal CA1 originates from neurons.^57^, ^58^ The origin of the elevated adenosine in the POA during noxious stimuli needs further exploration. On the molecular level, adenosine can be released through vesicular exocytosis or via nucleoside transporters. The exocytotic process occurs rapidly, possibly in less than 1 ms,^57^, ^59^ while our results showed an elevation in POA adenosine levels approximately 1 min after noxious stimuli. This discrepancy may suggest that, in our research, adenosine is not released through exocytotic mechanisms; however, this needs to be verified by more precise methods. Additionally, adenosine can also be derived from the degradation of extracellular ATP.^60^, ^61^ Investigation into the release mechanisms of adenosine is also of significant importance.

Further, we showed that adenosine A1 and A2A receptors in the POA might mediate the hypothermic response following nociceptive stimulation. Inhibiting adenosine A1 or A2A receptors notably attenuated the decrease in body temperature induced by astrocytic activation. Generally, A1 receptors govern inhibitory synaptic functions, while A2A receptors regulate excitatory synaptic functions.^62^, ^63^, ^64^ Considering adenosine's similar nanomolar affinities for A1 and A2A receptors,^65^ astrocytes may integrate the functions of excitatory and inhibitory synapses to induce hypothermia.

## 5 CONCLUSION

Our findings emphasize the pivotal role of POA astrocytes in integrating nociception and thermoregulation. When exposed to noxious stimuli, the activation of POA astrocytes orchestrates a reduction in body temperature, mediated by adenosine A1 and A2A receptors. This study expands our understanding of pain‐related physiological responses and contributes to elucidating their anatomical basis, which broadens our knowledge of changes in central nervous system function related to pain.

## AUTHOR CONTRIBUTIONS

Junke Jia: Conception and design, collection and assembly of data, data analysis and interpretation, and manuscript writing; Ting Chen: Conception and design, data analysis, manuscript writing, and financial support; Chang Chen and Tengxiao Si.: conception and design, data analysis, and interpretation; Chenyi Gao, Yuanyuan Fang, and Jiahui Sun: collection and assembly of data, and data analysis; Jie Wang: conception and design, manuscript writing, and supervision; Zongze Zhang: manuscript writing, supervision, and financial support.

## CONFLICT OF INTEREST STATEMENT

The authors have no conflicts of interest to declare.

## Data Availability

Data will be made available on request.
